# Advances and challenges in retinoid delivery systems in regenerative and therapeutic medicine

**DOI:** 10.1038/s41467-020-18042-2

**Published:** 2020-08-26

**Authors:** Raquel Ferreira, Joseph Napoli, Tariq Enver, Liliana Bernardino, Lino Ferreira

**Affiliations:** 1 Health Sciences Research Centre (CICS-UBI), Faculty of Health Sciences, University of Beira Interior, Covilhã, Portugal; 2 Nutritional Sciences and Toxicology, University of California, 231 Morgan Hall, MC#3104, Berkeley, CA 94720 USA; 3 UCL Cancer Institute, University College London, London, UK; 4 Center for Neuroscience and Cell Biology (CNC), University of Coimbra, Coimbra, Portugal; 5 Faculty of Medicine, University of Coimbra, Coimbra, Portugal

**Keywords:** Drug delivery, Materials science, Nanoscience and technology

## Abstract

Retinoids regulate a wide spectrum of cellular functions from the embryo throughout adulthood, including cell differentiation, metabolic regulation, and inflammation. These traits make retinoids very attractive molecules for medical purposes. In light of some of the physicochemical limitations of retinoids, the development of drug delivery systems offers several advantages for clinical translation of retinoid-based therapies, including improved solubilization, prolonged circulation, reduced toxicity, sustained release, and improved efficacy. In this Review, we discuss advances in preclinical and clinical tests regarding retinoid formulations, specifically the ones based in natural retinoids, evaluated in the context of regenerative medicine, brain, cancer, skin, and immune diseases. Advantages and limitations of retinoid formulations, as well as prospects to push the field forward, will be presented.

## Abstract (web summary)

Retinoids are involved in a wide range of cellular functions; as such, delivery systems for retinoids are of significant clinical interest. Here the authors review the advances in preclinical and clinical testing regarding retinoid formulations in the context of regenerative medicine, brain, cancer, skin and immune diseases.

## Introduction

**Fig. 1:**
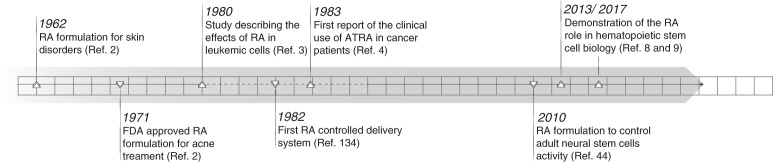
Milestones in RA formulations research. ATRA all-*trans* retinoic acid, FDA Food and Drug Administration, RA retinoic acid.

Retinoic acid (RA) signaling is one of the most important biological pathways in nature, triggered by RA interaction with nuclear receptors that control gene expression. The chemical structure of retinol (vitamin A, a RA precursor) was first described by Paul Karrer in 1931^1^, who was awarded a Nobel Prize in 1937 for the discovery. The use of RA for skin disorders^2^ and cancer treatment (acute myeloid leukemia (AML)^3^ and cervical neoplasia^4,5^) started in the 1960s and 1980s (Fig. 1). By the 2000s, RA had been incorporated in many tissue engineering scaffolds as a stem cell differentiation agent^6,7^. Over the last 10 years, many discoveries related to the biological role of RA in controlling the biology of hematopoietic stem cells^8,9^, tumor-initiating cells^10–12^, immune cells^13^, intestinal mucosa wound repair^14^, cancer resistance^15^, and cell reprogramming and differentiation^16,17^, have further stimulated the interest in this drug for many other biomedical applications. This interest is confirmed by more than 50 active clinical trials (according to ClinicalTrials.gov) evaluating the effect of RA in cancer (28 trials), mostly in hematological (16 trials) and brain tumors (8 trials), skin pathologies (e.g., acne, photoaging, eczema) (5 trials), and in other conditions such as inflammation, olfactory loss, and neuropsychiatric diseases (Table 1).

**Table 1:** RA formulations tested in past and ongoing clinical trials.

Drug	Indication	Clinical trial
ATRA-containing liposomes	Acne	Phase I/II (NA)^71^
ATRA-containing collagen sponge	Mild/moderate intraepithelial cervical neoplasia	Phase II (NA)^4,5^
ATRA + pembrolizumab	Advanced melanoma	Phase I/Ib (NCT03200847)
ATRA + ipilimumab	Advanced melanoma	Phase II (NCT02403778)
13-*cis* RA + cabozantinib	Solid tumors	Phase I (NCT03611595)
13-*cis* RA + temozolomide + thiotepa + carboplatin	Brain tumor	Phase II (NCT00528437)
13-*cis* RA + 3F8/GM-CSF	Neuroblastoma	Phase II (NCT01183429)
13-*cis* RA + 3F8/GM-CSF	Neuroblastoma	Phase II (NCT01183897)
13-*cis* RA	Neuroblatoma	Phase I/II (NCT03291080)
13-*cis* RA + dinutuximab + lenalidomide	Neuroblastoma	Phase I (NCT01711554)
13-*cis* RA + several drugs	Neuroblastoma	NA (NCT01526603)
ATRA	Cholangitis, sclerosing	Phase II (NCT03359174)
ATRA + arsenic trioxide	APL	Phase II (NCT01404949)
ATRA + arsenic trioxide	APL	Phase III (NCT02339740)
ATRA + arsenic trioxide + gemtuzumab ozogamicin	APL	Phase II (NCT01409161)
ATRA + idarubicin	APL	NA (NCT01064557)
ATRA + arsenic trioxide + realgar-Indigo naturalis formula	APL	Phase III (NCT02899169)
ATRA + several drugs	APL	Phase IV (NCT02200978)
ATRA + several drugs	APL	Phase III (NCT02688140)
ATRA + several drugs	APL	Phase III (NCT00482833)
ATRA	Acne vulgaris	Phase IV (NCT02620813)
ATRA	Multiple myeloma	Phase I/II (NCT02751255)
ATRA + rituximab	Immune thrombocytopenia	Phase II (NCT03304288)
ATRA + 5-azacitidine + lupron	Prostate cancer	Phase II (NCT03572387)
ATRA	Olfactory loss	NA (NCT03574701)
ATRA + tranylcypromine + cytarabine	AML	Phase I/II (NCT02717884)
ATRA + tranylcypromine	AML	Phase I (NCT02273102)
ATRA + gemtuzumab ozogamicin	AML	Phase III (NCT00893399)
ATRA + decitabine + cytarabine + G-CSF	AML	Phase II (NCT03356080)
ATRA + arsenic trioxide + cytarabine	AML	Phase I/II (NCT03031249)
ATRA + pioglitozone + azacitidine	AML	Phase II (NCT02942758)
ATRA + gemcitabine + Nab-paclitaxel	Pancreatic cancer	Phase I (NCT03307148)
ATRA + INCB059872 + azacitidine + nivolumab	Advanced malignancies	Phase I/II (NCT02712905)
13-cis RA + vorinostat + temozolamide	Glioblastoma	Phase I/II (NCT00555399)
Retinol + bakuchiol	Photoaging; wrinkles	Phase I/II (NCT03112863)
9-*cis* RA + cyclosporine A	Hand eczema	Phase III (NCT03026946)

AML, acute myeloid leukemia; APL, acute promyelocytic leukemia; ATRA, all-*trans* retinoic acid; GM-CSF, granulocyte-macrophage colony-stimulating factor; NA, not available.

Clinical applications of RA have highlighted three main limitations of its pharmacological use. First, RA is poorly soluble in aqueous solutions^18^ and photosensitive^19^, which makes its administration challenging. Secondly, RA induces irritation when applied onto the skin and increases its catabolism, when it is administered intravenously, reducing its therapeutic efficacy^20^. Lastly, RA is involved in many biological processes and thus the systemic delivery of RA causes side effects. All these limitations motivated researchers to synthesize novel and better tolerated synthetic retinoid compounds and to develop retinoid delivery formulations based on gels, liposomes, microparticles, nanoparticles, and micro-/nanofibers, which in some cases have been modified to target-specific tissues and cells of interest^21^.

Recent developments in the use of retinoid for cancer treatment^22,23^ and differentiation studies using stem/progenitor cells^8,24^, as well as the development of more advanced formulations to control retinoid bioactivity^25,26^, make this review timely. In addition, except for a limited number of reviews, with a restricted scientific scope in retinoid formulations (treatment of skin disorders)^27^ or highlighting the importance of retinoids in development (early organogenesis)^28^ or for specific therapeutic applications (cancer and metabolic disease)^29^, no study has fully covered the application of retinoid formulations for diverse therapeutic and regenerative medicine applications. In this review, the role of retinoid formulations in the context of the brain, skin, immune system, and cancer applications will be discussed. For each application, the pathological context will be briefly presented as well as the effect of retinoid that was administered without any controlled release system. The reasons behind the development of retinoid formulations in each application will be presented, along with their benefits and limitations, considering retinoid solubility, photostability, biocompatibility, release profile, tissue/cell availability and targeting, and therapeutic efficacy.

## Retinoid chemistry and general overview of RA signaling

### Retinoid chemistry

Retinoids are a class of compounds composed of three regions: a hydrophobic, a central polyene, and a polar (usually a carboxyl group)^29^. There are three natural retinoids: all-*trans* RA (ATRA), 9-*cis* RA (alitretinoin), and 13-*cis* RA (isotretinoin). To decrease the toxicity of the natural retinoids as well as to increase their stability and selectivity against a specific RAR subtype, several semi-synthetic and synthetic retinoids (including “atypical retinoids”) have been developed^29,30^. Some of them have been tested in clinical trials and approved for therapy (Table 2). At least four molecules have reached phase 4 clinical trials: bexarotene, tazarotene, adapalene, and trifarotene^29–31^. Bexarotene has optimal RXR binding, which effectively causes cancer cell death, particularly in cutaneous T-cell lymphoma. However, high levels of serum aminotransferase and liver injury have been reported^32^. Tazarotene and adapalene formulations have high affinity and selectivity for RARβ and RARγ, although the first one showed more toxicity than the second in dermatological applications^33^. Trifarotene is a selective RAR-γ agonist, approved in late 2019 in the USA, for the topical treatment of acne in patients as young as 9 years old^31^. Despite the progress made in the last years in the synthesis of novel retinoids to increase natural retinoid efficacy while reducing their toxicity, it is evident that natural retinoids are still under intense scrutiny as demonstrated by a considerable number of clinical trials (Table 1). The reasons are likely combinatorial: RA is a natural drug, blocks multiple disease signaling pathways^12^, in opposition to some synthetic retinoids that are very selective to a single receptor-mediated signaling pathway, and it has been used for many years in combinatorial therapy and thus well known by clinicians. Because it would be difficult to cover all advances made in retinoid delivery systems in a single review, the authors have chosen to cover only natural retinoids in the present paper. This focused approach is also supported by recent progresses in RA biology and the several formulations developed over the last years for the delivery of RA.

**Table 2:** Synthetic retinoids in terminated or active (in bold) clinical trials and approved for commercialization.

Drug	Receptor activity	Indication	Clinical trial
Tamibarotene (or Am80)	RARα agonist	Crohn’s disease	Phase II (NCT00417391)
		APL	Phase II (NCT00520208)
		Advanced nonsmall cell lung cancer	Phase I (NCT01337154)
		AML or myelodysplastic syndrome	**Phase II (NCT02807558)**
Palovarotene	RARγ agonist	Eye dry disease	**Phase I (CTP300302)**
		Fibrodysplasia ossificans progressive	**Phase III (NCT03312634)**
		Multiple osteochondromas	**Phase II (NCT03442985)**
Trifarotene (cream)	RARγ agonist	Moderate facial and truncal acne vulgaris	**Phase III (NCT03915860)**
		Autosomal recessive ichthyosis with lamellar scale	**Phase II (NCT03738800)**
		Early cutaneous T-cell lymphoma	Phase I (NCT01804335)
Bexarotene (capsules)	Pan agonist	Refractory cutaneous T-cell lymphoma	Approved by FDA since 1999
Tazarotene (gel or cream)	Pan agonist	Hand–foot skin reactions	**Phase II (NCT04071756)**
		Facial acne vulgaris	Approved by FDA since 1997
		Plaque psoriasis	Approved by FDA since 1997
Adapalene (solution, cream, and lotion)	Pan agonist	Acne	Approved by FDA since 1996
AGN194204	RXR agonist	Prostate cancer	Phase II (NCT01540071)
UAB30/9-cis-UAB30	RXR agonist	Nonmelanoma skin cancer	**Phase I/II (NCT03327064)**
Fenretinide (oral powder and intravenous liquid emulsion)	Atypical retinoid	Peripheral T-cell lymphoma	**Phase II (NCT02495415)**
		Solid tumor (relapsed malignancies)	**Phase I (NCT01553071)**
		High risk cancer	Phase III (NCT01479192)
		Prevention of bladder cancer	Phase III (NCT00004154)
		Cervical neoplasia	Phase III (NCT00003075)
		Schizophrenia	Phase III (NCT00534898)
		Breast cancer	**Phase III (NCT01357772)**

AML, acute myeloid leukemia; APL, acute promyelocytic leukemia; FDA, Food Drug Administration; RAR, retinoic acid receptor; RXR, retinoid X receptors.

### RA signaling

**Fig. 2:**
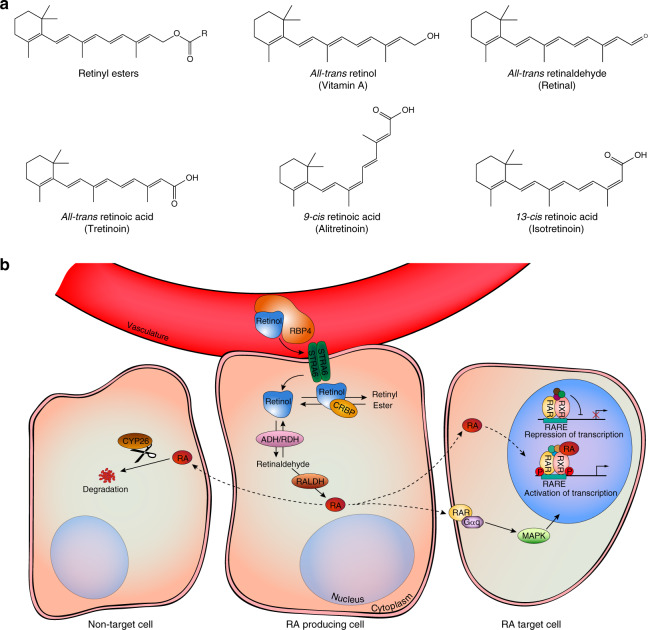
Retinoid chemical structures (**a**) and RA signaling pathway (**b**). Blood-circulating retinol is internalized through membrane transporter and receptor *stimulated by retinoic acid 6* (STRA6) and converted into all-*trans* retinoic acid (RA), which binds to cellular retinoic acid-binding protein type 2 for signaling in the nucleus. RA triggers gene transcription by binding to RA receptors (RAR) and to the retinoid X receptor (RXR). In the presence of the ligand, RAR and RXR heterodimerize on retinoic acid-response element (RARE) sequences located in promoter regions inducing the transcription of target genes. Of note, RXR may also homodimerize and trigger gene transcription (not depicted in the illustration). RA signaling may also occur via activation of receptors associated with lipid rafts located on the cell surface, which trigger transcriptional activation of target genes by histone and receptor phosphorylation in the cell nucleus. ADH alcohol dehydrogenase, CRBP cellular retinol-binding protein 1, CYP26 cytochrome P450 family 26, Gαq Gq protein alpha subunit, MAPK mitogen activated protein kinases, P phosphorylation, RALDH retinaldehyde dehydrogenase, RBP4 retinol-binding protein 4, RDH retinol dehydrogenase.

RA is the main biologically active metabolite of vitamin A^28^. In humans, the only source of vitamin A is obtained through diet, as lipophilic retinol (or its more stable form, retinyl ester) or as carotenoids. The transport of these retinoids to cells occurs when blood-circulating retinol is bound to retinol-binding protein (RBP) 4 (Fig. 2). This complex interacts with membrane transporter and receptor *stimulated by retinoic acid 6* facilitating entry into the cytoplasm, where retinol binds to Crbp1 (encoded by RBP1). A two-step process converts retinol into ATRA. RA binds to cellular retinoic acid-binding proteins (CRABP), assisting autocrine and paracrine signaling^34^. The mechanisms underlying paracrine signaling remain unclear while autocrine signaling requires CRABP2 for nuclear entry^28,34^. In the nucleus, RA triggers gene transcription by binding to heterodimers formed by RA receptors (RARα, RARβ, and RARγ) and retinoid X receptors (RXRα, RXRβ, and RXRγ). RAR–RXR heterodimers interact with a deoxyribonucleic acid sequence known as the retinoic acid-response element^35^, which facilitates the binding of co-activators to histone acetylase, ultimately leading to the transcription of target genes (Fig. 2). Recent data showed that RXRα homodimers can also regulate gene transcription^36^. Another isomer of RA, isomer 9-*cis* RA (alitretinoin), binds to retinoid X receptors^28,35^, while 13-*cis* RA (isotretinoin), has negligible affinity for retinoic acid receptors (RAR or RXR) or cellular RBP^37^. However, 13-*cis* RA may be converted into molecules that act as agonists for nuclear RAR and RXR. Importantly, RAR also regulates nonnuclear and nontranscriptional effects, namely the activation of kinase signaling pathways^38^ (Fig. 2). Finally, RA is catabolized by monooxygenases of the cytochrome P450 superfamily^34^.

## Formulations for the delivery of RA

RA has very low solubility in aqueous solution (0.21 μM at pH 7.3)^18^ and thus requires specific binding proteins (e.g., CRABPs) to be transported within cells to act at nuclear receptors. In addition, it has a short (few hours) lifetime, due to its degradation by a cytochrome P450-dependent monooxygenase system^39^. Moreover, it induces undesirable side effects (congenital malformations^40^ as well as mucocutaneous dryness, headache, and hypertriglyceridemia^41^) when administered at high concentrations. Therefore, for more than 35 years, several groups have developed different RA delivery systems to overcome these limitations. Delivery systems based in polymeric scaffolds (e.g., hydrogels, nanofibers), nanoparticles (liposomes, micelles, polymeric, dendrimers), microparticles, among others, are described and summarized in Table 3.

**Table 3:** Examples of formulations for the controlled release of RA in the context of cancer, brain diseases, skin diseases, immune diseases, stem cell differentiation, among other applications.

Type	Diameter	Loading (μg/mg of carrier)	Delivery	Applications	Ref.
NPs	≃500 nm	≃76	Up to 3 h in the presence of trypsin	Pharmaceutical	^94^
NPs	<200 nm	154	Release up to 10 days	Cancer	^51^
NPs	170–230 nm	20	80% cumulative drug release in 4 days	Immune diseases	^58^
NPs	170–185 nm	275	Sustained drug release observed over 72 h	Cancer	^56^
NPs	≃200 nm	86	17% cumulative RA release for 21 days	Stem cells and brain diseases	^24,44,66^
NPs	160 nm	150	50% release after 10 min irradiation	Cancer and brain diseases	^25,60^
NPs	≃100 nm	68–87	5–100% release of RA in 5 days	Stem cells and brain diseases	^45^
NPs	≃214 nm	30	38% cumulative release of RA in 48 h	Cancer	^120^
MPs	≃5.6 μm	80	Pseudo-zero order release for 5 weeks	Pharmaceutical	^61^
MPs	≃8 μm	3	Release up to 10 days	Stem cells	^7^
MPs	190 μm	57	Less than 3 h	Cancer	^134^
Mic	100–500 nm	40–130	NA	Pharmaceutical	^48^
Mic	100–400 nm	2.6	Parenteral administration; <5% in 3 days	Cancer	^63^
Mic	50–200 nm	80% (w/w)	1-month delivery	Cancer	^47^
Lip	NA	NA	4.4 μg/mL blood per day (in humans)	Cancer	^49,70,95^
EFM	0.95–1.89 μm	5	80% cumulative RA release in 3.5 months	Stem cells	^135^
EFM	<5 μm	≃10	≃9.0 μM for 1 h	Stem cells	^73^
Scaffold	NA	≃20	Release over 8–28 h	Regenerative medicine	^136^
Scaffold	0.1–0.85 μm fibers	≃0.3–7	Sustained release of RA for up to 1 week	Stem cells	^137^

EFM, electrospun fibrous mesh; h, hour; Lip, liposome; Mic, micelles; MPs, microparticles; NA, not available; NPs, nanoparticles; min, minute.

### Type and strategies for the delivery of RA

Several strategies have been used to prepare RA delivery systems: namely, by complexation of RA with proteins (e.g., transthyretin)^42^ or cationic polymers (e.g., poly(ethyleneimine) (PEI))^43,44^, by physical encapsulation in polymeric^25,44^ or inorganic^45^ nanoparticles, microparticles^46^, micelles^47,48^, liposomes^49^, or films^43^, by covalent attachment of RA to a carrier^50^, or by immobilization of RA on surfaces of nanoparticles^45^, among others. Despite several reports on RA delivery systems based on polymeric scaffolds or electrospun fibrous meshes, the most common strategy has been based on liposomal or polymeric nanoparticles formed by polyesters, polyimines, polysaccharides, and proteins (Table 3). A significant number of formulations have allowed the RA encapsulation in the core of the nanoparticles. This RA encapsulation was either obtained by (1) physical or (2) chemical interaction with the components of the nanoparticle or (3) by physical entrapment. Regarding physical interaction, RA is complexed with positively charged polymers such as chitosan, or PEI^43,51^. The carboxylic acid of RA interacts electrostatically with the amine group located in the polymer, forming a complex that can be stabilized by addition of a polyanion and divalent ions^43,44^. Concerning chemical interaction, RA is typically conjugated chemically to one of the components of the nanoparticle by biodegradable ester^50,52^, amide^26,53–55^, or disulfide^56^ linkages. These bonds are susceptible to degradation during specific pH conditions, in the presence of proteases, or reducing agents that lead to RA release. Concerning physical entrapment, RA is captured during nanoparticle formation^57,58^ or in the pores of the nanoparticles^59^. In a relatively low number of formulations, RA was immobilized not in the core but on the surface of the nanoparticle^22^; however, the concentration of RA immobilized on the surface is lower than in the core. Formulations with high (above 100 μg/mg of formulation)^51,56,60^, medium (between 100 and 25 μg/mg of formulation)^44,45^, and low (below 25 μg/mg of formulation)^7,58^ loading have been described.

The release profile of RA depends on several parameters of the formulation, including the size, composition, initial concentration of loaded RA, and its degradation profile. The sustained release of RA for more than 1 month can be accomplished by encapsulating it into biodegradable microspheres and tuning the release rate by adjusting polymer composition in the formulation^61^, by the encapsulation in polyion complex micelles^62^ and adjusting polymer composition or drug content, or by the encapsulation in liposomes^49^. In general, formulations with high RA loading show a slower release profile of the drug^51^. Because of the hydrophobicity of RA, the occurrence of a burst release in most formulations is negligible^44,47,51,63^.

Recent developments to improve the delivery of RA have led to the design of formulations that can be controlled remotely (temporally and spatially) by an external stimulus such as light, ultrasound, or magnetic forces^64^. These stimuli-responsive biomaterials are suitable for controlling the kinetic of RA delivery. In that sense, light-activatable nanoparticles containing RA that disassemble in minutes after activation by a blue laser at 405 nm have been prepared^25,26,60^. Importantly, these formulations presented higher activity than formulations in which RA was released by passive diffusion (not light triggered) because they rapidly saturated nuclear receptors.

### Ability of RA formulations to cross biological barriers

The cellular uptake of some RA-containing nanoparticles has been demonstrated to take <12 h^44^ by clathrin-mediated endocytosis and macropinocytosis^25^. Formulations that escaped the endolysosomal compartment accumulated in the cytoplasm in less than 24 h^25,45^. RA released in the cell cytoplasm may bind to transport proteins such as cellular retinoid-binding protein II (CRABP-II) and/or fatty acid-binding protein 5, followed by its transportation to the cell nucleus^65^. The intracellular concentration of formulations containing RA was dependent on the initial formulation loading, type of formulation, and type of cell^25,45^. Uptake between 25 and 80 pg of nanoparticles containing RA per cell has been described^25^.

The capacity of RA-containing formulations to cross biological barriers such as the blood-brain barrier (BBB) (relevance for the treatment of brain cancer and neurodegenerative disorders) is a topic largely unexplored. In most cases, the formulations have been administered by stereotaxic and not intravenous injection^66^. Experimental data in humans indicate that ATRA administered orally is not able to accumulate in the cerebrospinal fluid^67^. Clinical trials such as NCT00528437 (Table 1) and others are now investigating the pharmacokinetics of 13-*cis* RA and its accumulation in the cerebrospinal fluid.

### Preclinical and clinical uses of RA formulations

**Fig. 3:**
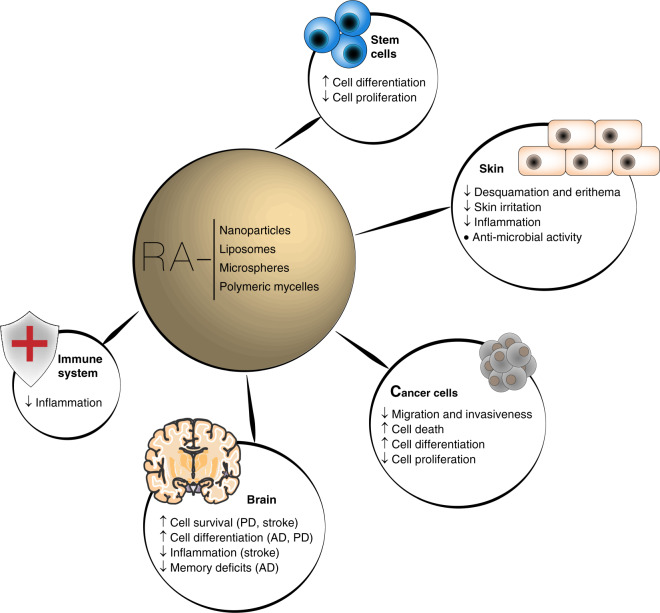
Main outcomes of RA-based therapies in pathological contexts. AD Alzheimer’s disease, PD Parkinson’s disease, RA retinoic acid, ↑ = increased effect, ↓ = decreased effect, ● = regulatory effect.

Formulations containing RA have been used both in preclinical and clinical trials (Fig. 3). Most preclinical tests were performed in mice in the context of regenerative medicine^45,60,66,68^ and cancer^22,25,56^. In vitro tests showed that ATRA-containing liposomes^69^ or ATRA-containing polymeric nanoparticles^25,44^ were 100–1000 times more active than soluble ATRA in cultured tumor cells^25,69^ or neural stem cells (NSC)^44^. In vivo tests showed that rats^49^ or human patients^70^ treated with ATRA-containing liposomes by intravenous administration had no decrease in plasma ATRA levels while animals/humans treated with oral formulation of ATRA (nonliposomal) had a significant decrease in plasma ATRA levels^41,49^. The results indicate that the hepatic metabolism of ATRA encapsulated in liposomes was inferior to the one observed in ATRA administered orally. Both formulations were safe in human trials. The clinical application of RA-containing formulations can be divided into two groups: (1) topical and (2) oral administration. For topical administration, seven RA-containing formulations have reached the market for the treatment of skin-related diseases (Table 4). Here, current progresses are concentrated in reducing the toxicity of RA (e.g., by the use of synthetic retinoids^31^), and exploring the combination of RA with other drugs (Table 4). For oral administration, four formulations containing RA have reached the market, particularly for the treatment of skin diseases (three of them) and cancer (only one) (Table 4). Others were tested in clinical trials but did not reach the market. For example, a liposomal-based formulation of RA (monotherapy) was successfully tested in patients with facial acne^71^ and refractory hematological malignancies in a phase II clinical trial^70^. In the last application, the remission rate (67% clinical remission) was lower than that obtained using a combinatorial therapy (77% clinical remission), which included oral administration of ATRA plus idarubicin and thus the liposomal formulation was not further evaluated. Unfortunately, the liposomal formulation of RA was not tested in combination with other drugs, as it is now being investigated with nonliposomal RA formulations (Table 1), and this deserves further investigation in the near future.

**Table 4:** Marketed RA formulations.

Name/company	Composition	Indication	Year approved	Ref.
9-*cis* RA (brand name: Panretin)/Eisai Inc.	Topical formulation; 0.05% or 0.1% gel containing alitretinoin	Cutaneous Kaposi’s sarcoma; treatment of recalcitrant chronic hand dermatitis	2000	^138,139^
13-*cis* RA (brand name: Accutane)/Roche	Topical formulation; 0.05% and 0.1% cream	Photoaging and acne	1982	^139^
ATRA	Topical formulation; 0.05% cream	Photoaging and acne	1971	^2^
Gel microsphere formulation containing ATRA/Advanced Polymer Systems	Topical formulation; macroporous beads, 10–25 μm in diameter	Acne	1997	^75,140^
9-*cis* RA	Topical formulation; 0.1% gel	AIDS-related Kaposi’s sarcoma	1999	^75^
Retinol	Topical formulation; 0.5–5% lotion, cream	Cosmetic	NA	^75^
Retinaldehyde	Topical formulation: 0.01, 0.015, 0.1% cream	Cosmetic	NA	^75^
ATRA (brand name: Vesanoid)/Roche	Oral formulation of tretinoin	Acute myeloid leukemia, in particular, acute promyelocytic leukemia	2000	^132^
Acitretin	Oral formulation	Psoriasis, disorders of keratinization	1997	^141^
13-*cis* RA	Oral formulation	Severe acne/related disorder	1982	^75^
Retinol	Oral formulation	Prevent/treat hypovitaminosis A	NA	^75^

AIDS, acquired immunodeficiency syndrome; ATRA, all-*trans* retinoic acid; NA, not available.

## RA delivery systems for regenerative medicine

### Embryonic stem cells

One of the initial applications of RA for regenerative medicine was as a differentiation agent during embryogenesis. The spatiotemporal release of RA by polymeric microparticles incorporated within embryonic bodies, derived from human embryonic stem cells, was reported to induce cell differentiation and tissue formation resembling the phenotype and structure of early human embryos^7^. Initial studies have used RA as a potent regulator of neural differentiation^72^. RA downregulates expressions of geminin and zinc finger protein Zic2, SoxB1 (Sox-1, Sox-2, Sox-3), and Notch-1, which maintain neural progenitor cell proliferation. By halting proliferation, RA shifts signaling toward differentiation. Several platforms have been used for RA delivery alone or in combination with other agents. For example, RA-containing electrospun fibrous meshes and scaffolds reportedly have an improved effect on stem cell dynamics. Electrospun poly(ε-caprolactone) fibers were loaded with ATRA, which led to extremely high local concentrations of this agent and the differentiation of murine embryonic cells, within the multilayered scaffolds^73^. Besides the neurogenic potential of RA, recent studies have highlighted the critical role of RA in the derivation of embryonic hematopoietic cells^9^, lymphoid organs^13^, and Langerhans cells that reside specifically in the epidermis^16^. Particularly, ex vivo activation of RA signaling in hemogenic endothelium, a small subpopulation of endothelial cells that can differentiate into hematopoietic cells, increased its transition into a pool of hematopoietic stem cells. Conversely, RA pathway shutdown terminated this process^9^. In addition, fetal type 3 innate lymphoid cells (ILC) are modulated by RA signaling in utero and therefore depend on appropriate maternal dietary intake of retinoids throughout pregnancy. This period also determines the ability of ILC progenitors to differentiate into mature lymphoid tissue-inducing cells^13^. It is possible that some of the RA delivery systems developed so far may be used for embryonic immune and hematopoietic stem cell development studies.

### Adult stem cells

RA is also an important regulator of adult stem cells. For example, ATRA antagonizes stress-induced activation of dormant hematopoietic stem cells by restricting protein translation and oxidative stress^8^. When mice were fed a vitamin A-free diet to deplete the RA reservoir, animals suffered, among other effects, functional impairment of hematopoietic stem cells and their numbers were unable to recover even after injection with an immunostimulant^8^. In addition, RA-based formulations have been used as inflammatory modulators of stem cells. For example, human mesenchymal stem cells exposed to ATRA-loaded solid lipid nanoparticles significantly reduced IL-6 and IL-8 expression^74^. ATRA-containing nanoparticles have been also developed to deliver RA into NSC niches^44^. The nanoparticles had a higher effect on neuronal differentiation than solubilized RA both in vitro and in vivo^44,66^. The effect was mediated by an increase in transcription of the pro-neurogenic genes Ngn1 and Mash1^44,66^. The formulation was then modified to remotely disassemble and release RA with spatial and temporal resolution, triggered by exposure to blue light^60^. A single short pulse of light prompted β-catenin-dependent neuronal differentiation and RARα upregulation. The combined action of blue light and RA enhanced endogenous neurogenesis.

## RA delivery systems for skin diseases

### RA mode of action

Several RA drugs are clinically available for dermatological treatments, including ATRA and 9-*cis*-RA, among others^75^. For example, 9-*cis*-RA encapsulated in a gel is an FDA-approved topical agent for cutaneous Kaposi’s sarcoma^75^. ATRA has also been tested in clinical trials for the treatment of the same disease^76^. This sarcoma is associated with human immunodeficiency virus infection and is characterized by a vascular endothelioma (i.e., tumor of the endothelial cells). ATRA is used clinically for treating photoaging, acne, and psoriasis. Indeed, ATRA was approved for acne vulgaris treatment in 1971^2^, and since then, other drugs (called retinoids because they bind to RAR and/or RXR receptors) have been developed. In these clinical applications, the biological effect of ATRA includes: (1) modulation of proliferation and differentiation of skin cells; (2) anti-inflammatory activity^75^. The existence of several types of receptors and their combinations as heterodimers, as well as the ability of ATRA to modulate the activity of multiple kinase signaling pathways independently of the nuclear activation of RAR and RXR receptors, may explain the diversity of ATRA biological actions^35^. ATRA also induces skin angiogenesis and collagen deposition, increases the mitotic activity of inter- and follicular epithelium, and reduces melanin production^75^.

### Type of RA-containing formulations

**Fig. 4:**
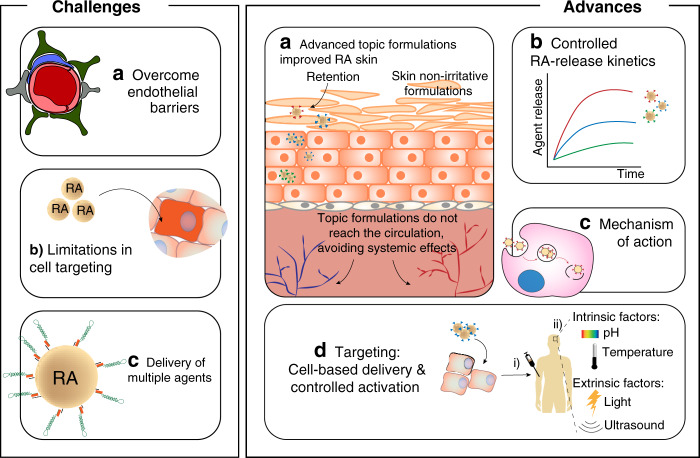
Challenges and advances in topical and systemic administration of RA-containing formulations. Challenges include: **a** crossing endothelial barriers for extravasation of RA-containing formulations into a specific body region; **b** targeting of RA-containing formulations to specific cells; **c** development of formulations that combine RA with other pharmacological agents, able to release each agent with a specific release kinetics. Advances include: **a** development of formulations with less toxicity; **b** release of RA with variable release kinetics to achieve variable biological actions; **c** action mechanism of RA during development and disease; **d** use of cells to transport RA-containing formulations to specific regions in the body followed by the triggering of the formulations by intrinsic (e.g., temperature, pH) or extrinsic (e.g., ultrasound, light) stimuli. RA retinoic acid.

Conventional formulations containing ATRA require multiple applications to maintain the therapeutic effect. Therefore, several formulations have been developed to improve the long-term effect of ATRA, to reduce side effects like desquamation and erythema, skin irritation, and to increase the stability of RA to light^19^ (Fig. 4). In this sense, formulations including phospholipid-based particles (e.g., solid lipid nanoparticles and nanostructured lipidic carriers)^2,77^, polymeric nanoparticles^2,27,77,78^, or polymers conjugated with RA^52^ have been developed to overcome these issues. The carriers presented a particle size between 100 and 400 nm, a range of zeta potential from neutral (liposomes) to negative (polymeric nanoparticles, ethosomes, solid lipid nanoparticles, and nanostructured lipidic carriers), high entrapment efficiency (above 65%), high photostability (between two- and threefold higher than commercial tretinoin dissolved in ethanol), moderate to high skin permeation, high skin tolerance, and moderate to high antipsoriatic activity. Some of these formulations are easier to scale-up (e.g., solid lipid nanoparticles) than others^77,79^. In addition, some formulations (e.g., ATRA-containing liposomes) improve the local effect of RA in the skin and decrease systemic adsorption^78^. Moreover, ATRA-containing formulations increased significantly the chemical stability of ATRA during normal storage conditions (e.g., stability for 1 year at 25 °C) and after exposure to UV irradiation (twofold lower photodegradation)^77,80^. The protective effect of these formulations was linked to their ability to reflect and scatter UV radiation^80^. Interaction of ATRA with a lipophilic amine (stearylamine) decreases ATRA crystallinity, leading to a formulation with less skin irritating properties^77^.

### Preclinical and clinical applications

RA-containing formulations are under clinical evaluation for the treatment of *acnes vulgaris*, hand eczema and photoaging (Tables 1 and 3). An ATRA-loaded liposomal formulation has been tested in a pilot trial^71^. Patients with facial acne treated with the liposomal formulation showed higher lesion improvements than with conventional formulations. The higher efficacy of the ATRA-loaded liposomal formulation was attributed to enhanced penetration of RA across the *stratum corneum*. A commercial tretinoin gel microsphere formulation consisting of microspheres with 10–20 μm in size^75^ has reached the market. A common issue in designing controlled RA release systems for skin applications is the limited efficacy in terms of cell targeting (Fig. 4). Formulations such as micro- and nanoparticles might have the capacity to target particular cells by specificities in their physicochemical properties or by incorporating in their surface peptides, proteins, or aptamers that recognize specific cell receptors. Due to their size, these formulations might accumulate preferentially in some regions of the skin, such as the follicular adduct and thus act in those biological environments. Indeed, follicular targeting is important for the treatment of acne because it increases the therapeutic effect of retinoids, while reducing their potential side effects. Polymeric micelles of diblock methoxy-poly(ethylene glycol)-poly(hexyl-substituted lactic acid) copolymer favor follicular aduct-targeted delivery of ATRA^81^.

## RA delivery systems for brain diseases

Several formulations containing RA have been used to treat Alzheimer’s disease (AD), Parkinson’s disease (PD), and stroke (Table 1). Current challenges in the use of RA formulations for brain diseases are ascribed to low accumulation in the brain after intravenous administration (Fig. 4). Although the formulations having affinity ligands in their surface have increased retention in the brain, only a minor fraction of the injected formulation reaches the brain and crosses the BBB. Cell-mediated delivery (e.g., T cells, monocytes) may be used to overcome this issue: cells can act as transporters of formulations that will be activated by local (e.g., pH) or remote triggers (e.g., light, ultrasound, magnetism)^25,82^ once they reach the target site.

### Alzheimer’s disease

AD is a neurodegenerative disease caused by the accumulation of amyloid-beta peptides, hyperphosphorylated tau filaments, and brain vascular changes leading to cerebral amyloid angiopathy^83^. In experimental models of AD, RA: (1) inhibited oxidative damage and mitochondrial dysfunction; (2) increased ApoE expression and suppressed inflammation; and (3) improved learning and memory^84,85^. Nanoparticles have been developed to efficiently deliver ATRA and small interfering RNA to promote NSC differentiation in AD^45^. In this study, the transplantation of nanoparticle-treated NSCs in AD mice improved cognition and memory.

### Parkinson’s disease

PD is characterized by selective degeneration of dopaminergic neurons in the *substantia nigra* and by the accumulation of α-synuclein and Lewy bodies (protein inclusions in neurons)^68^. In experimental models of PD, RA protected against neurodegeneration of midbrain dopaminergic neurons in the *substantia nigra*^86,87^. Stereotaxic injection of ATRA-containing nanoparticles in the striatum protected nigral dopaminergic neurons in an in vivo PD model^68^. Accordingly, RA-containing nanoparticles increased expression of Nurr1 and Pitx3, key regulators of dopaminergic neuronal development and maintenance^68,88^.

### Stroke

In the case of stroke, changes in the vasculature, or BBB permeability or function, may cause or enable the progression of CNS diseases^89^. RA/RAR signaling is critical for BBB differentiation and integrity^90^. Recent studies have shown protective effects of ATRA-containing nanoparticles in stroke. The formulation enhanced endothelial cell proliferation and tubule network formation and protected against ischemia-induced death in endothelial cell lines and endothelial progenitor cells isolated from ischemic stroke patients^24^. Moreover, when intravenously injected, RA formulations restored neuronal and vascular functions in a prenatal model of brain ischemia^91^.

## RA delivery systems for the treatment of cancer

### Blood cancers

*RA mode of action*: one of the first applications of RA delivery systems was for the treatment of blood cancers. The antitumor activity of ATRA was demonstrated in 1980 in acute promyelocytic leukemia (a subtype of AML accounting for 5% of AML cases)^3^. Since then, many clinical trials have evaluated the antitumoral efficacy of the drug alone or in combination with arsenic trioxide or idarubicin^92^. RA promoted terminal differentiation of leukemic cells while reducing their proliferation. The antitumoral activity of RA (enhanced by cooperation with arsenic trioxide)^11^ was due to inhibition and degradation of prolyl isomerase Pin1, which has a critical role in coordinating multiple phosphorylation events during oncogenesis^12^. Therefore, RA has the unique property of blocking multiple cancer-driving pathways simultaneously. Unfortunately, the therapeutic efficiency of ATRA-based therapies remains to be demonstrated in patients with AML without acute promyelocytic leukemia.

*Type of RA-containing formulations*: physicochemical properties as well as release properties of RA-containing formulations for the treatment of blood cancers are summarized in Table 3. With the exception of some formulations for parenteral administration^63^ or for intracellular delivery in ex vivo conditions^25,26^, most RA-containing formulations have been developed for intravenous delivery. These formulations were based in liposomes^49,70,93^, microspheres^61^, polymeric micelles^63^, and nanoparticles^50,94^. In most cases, ATRA was encapsulated in the formulation^49,70^, or chemically conjugated to the carrier^50^. This effort was motivated by the fact that some of the patients treated with ATRA relapsed during the 4–6 weeks treatment of daily oral administration. Follow-up studies indicated that resistance was due to the decrease of ATRA concentration in the plasma, and thus the inability of the drug to differentiate leukemic cells^41^. The decrease was attributed to an induction of ATRA catabolism and increased levels of RA-binding protein^20^. Several ATRA delivery systems showed relative success in reducing the induction of ATRA catabolism and thus maintaining RA for longer periods in blood plasma. For example, microspheres of poly(l-lactide) showed a nearly constant release rate of ATRA for 5 weeks^61^. In addition, intravenous administration of RA-containing liposomes for 7 weeks in rats did not enhance RA catabolism^49^. Maintenance of RA in the plasma, via liposomal-encapsulated ATRA, reduced the number of relapses^95^.

Targeted delivery of RA-containing formulations to the bone marrow has recently attracted much attention^96^. Bone marrow is the residence of hematopoietic stem cells that give rise to myeloid and lymphoid cell lineages. In leukemia patients, hematopoietic stem cells are the target of genetic mutations and thus aberrant activity. These altered cells are difficult to eliminate by conventional antitumoral agents because drugs do not reach the bone marrow at the required concentration, and because the stem cell niche (a physical and functional entity that supports the self-renewal and differentiation of stem cells) changes/adapts after chemotherapy, protecting altered cells^97^. RA is a potential treatment, combined with other drugs, for AML^98^ and chronic myeloid leukemia^99^. In the last 10 years, a new therapeutic paradigm has emerged based on the targeted delivery of formulations to bone marrow^96^. Targeted delivery can be achieved by surface modification of nanoparticles with bisphosphonate to promote binding to bone^100^, by surface modification of liposomes with folate to target the folate receptor, which is highly expressed by AML cells^21,101^, by the conjugation of nanoparticles with the surface of hematopoietic stem cells^102^, or by surface modification of nanoparticles with antibodies (e.g., CD45.1, CD117)^103,104^ or aptamers (E-selectin thioaptamer)^105^. Recently, we have developed a new platform to target bone marrow, based on light-activatable nanoparticle formulations containing ATRA^25,26^. The formulation was highly internalized by leukemia-initiating cells and accumulated in the cell cytoplasm. Once leukemia-initiating cells transfected with light-activatable nanoparticles containing RA were administered intravenously in leukemic mice they tend to home in the bone marrow, in the proximity of other leukemia cells. The irradiation of bone marrow with a blue light induced photo-disassembly of the nanoparticles within the cells and consequently their differentiation. These cells then secreted extracellular vesicles containing ATRA that interfered with cells supporting the stem cell niche.

*Preclinical and clinical applications*: most RA-containing formulations are under clinical evaluation for the treatment of blood tumors (Table 1). Current challenges for translation of RA formulations to the clinic are related to two factors: (1) capacity to incorporate multiple drugs besides RA; (2) efficiently target hematopoietic cells (Fig. 4). In most cases, RA-based monotherapies are less efficient in interfering with tumorigenesis and cancer progression than combination therapies^29^. The efficiency of combination therapies is likely ascribed to several factors: (1) a combination of drugs may act at different sites of the antiproliferative signaling pathway and thus be more effective in the inhibition process; (2) combination therapies may decrease the probability of cancer resistance; (3) synergies between the drugs reduces the dose necessary for therapy, as well as their toxicity and treatment time. Currently, several clinical trials are investigating the combination of ATRA with arsenic trioxide, with arsenic trioxide and gemtuzumab ozogamicin (a monoclonal antibody against CD33 conjugated with ozogamicin, a cytotoxic agent), with epigenetic regulators such as tranylcypromine, with decitabine, cytarabine and granulocyte-stimulating factor, among others, in the context of AML (Table 1). Unfortunately, most RA formulations tested so far for blood cancers have not explored the simultaneous controlled release of RA and other agents. Thus, further investigation is needed to address this issue. It should be noted that a liposomal formulation having two agents (non-RA drugs) has been approved recently for AML^106^. Another important challenge in clinical translation of RA formulations is targeting. Preclinical tests have addressed different leukemia cell targets that have not yet been explored in clinical trials. The recent re-approval of gemtuzumab ozogamicin^106^ may inspire new approaches for RA formulation targeting.

### RA delivery systems for the treatment of solid tumors

*RA mode of action*: one of the first applications of RA formulations for the treatment of solid tumors was in mild/moderate intraepithelial cervical neoplasia^4,5^. ATRA was released by a collagen sponge with a cervical cap at the tumor site by insertion in the cervix. A dose of ∼0.4% of RA was selected for a phase II trial. Fifty percent of the patients showed a total regression of the disease^4,5^. Systemic and cervical side effects were mild and vaginal side effects were moderate and tolerable. Unfortunately, the formulation did not reach the market, likely because it was not sufficient to reverse or suppress more advanced dysplasia with acceptable local side effects in phase III clinical trial^107^. The antitumoral activity of RA is linked to its differentiation and cell growth arrest properties^108^. RA has been tested in several clinical trials and the results showed that RA alone did not present significant antitumoral activity against breast cancer^109^; however, when RA was combined with tamoxifen or paclitaxel it showed moderate antitumoral activity^110,111^.

*Type of RA-containing formulations*: in the last years, significant effort has been made by the scientific community to develop formulations able to release RA and other antineoplastic drugs. For example, nanoparticles containing both ATRA and paclitaxel (to inhibit cell division because the cell cannot disrupt the polymerized tubulin for cell division) had superior efficacy than formulations containing only paclitaxel^53^. In addition, pH-sensitive nanoparticles have been designed to release all-*trans* retinal and doxorubicin in weakly acidic tumors (pH 6.5) or acidic intracellular environments such as endosomes/lysosomes (pH 4.5–5.5)^112^. All-*trans* retinal was chemically conjugated to the nanoparticle polymer by hydrazone bonds that were labile under acidic conditions. Compared to free drugs, the nanoparticle formulation increased the accumulation of the all-*trans*-retinal and doxorubicin at the tumor site and induced higher levels of cell senescence and antitumoral activity. Progress has also been made in targeting cancer-initiating cells, which are resistant to chemotherapy and associated with tumor recurrence. ATRA liposomes and nanoparticles encapsulating simultaneously ATRA and doxorubicin have been used successfully to arrest the proliferation of breast cancer-initiating cells and to differentiate them^113,114^. In a combinatorial approach, RA differentiated cancer-initiating cells, whereas antineoplastic agents, such as doxorubicin, killed noncancer-initiating cells.

*Preclinical and clinical applications*: the results of several clinical trials indicate that 13-*cis* RA, alone or in combination, seems to be effective at different levels against malignant gliomas^115^, and cancer occurring in the central and peripheral nervous system^116^. Moreover, ATRA inhibits proliferation of glioblastoma cells in vitro and in vivo, while promoting their differentiation^117,118^. At high concentrations, ATRA may induce cell apoptosis^119^. RA-coated solid lipid nanoparticles showed higher in vitro toxicity relatively to glioblastoma cells than the parent drug^120^. In addition, polymeric micelles composed by methoxy-grafted chitosan and encapsulating ATRA were more effective at inhibiting glioblastoma cell line migration in vitro than the free agent^62^.

The immune system plays an important role in modulating tumor progression; however, limited infiltration of immune cells in the tumor site, as well as the existence of immunosuppressive agents, hamper their biological role. Therefore, a combination of chemotherapy with immunotherapy might be a better strategy to fight tumor biology. Indeed, immunotherapy may increase the sensitivity of cancer cells to chemotherapy and thus reduce its side effects^121^. Recently, a chemo-immunotherapy approach for melanoma was developed based on biodegradable hollow mesoporous nanoparticles containing three drugs: doxorubicin, ATRA, and interleukin (IL)-2^23^. IL-2 is a T-cell growth factor and can thus facilitate proliferation and activation of T cells. Animals treated with formulations containing the three agents were the ones with the most effective tumor growth inhibition and decreased metastasis. ATRA was effective in differentiating myeloid-derived suppressor cells, and in synergy with doxorubicin, contributed to increase the number of dendritic cells at the tumor site; IL-2 facilitated the proliferation of CD8^+^ T cells at the tumor site to promote effective tumor killing.

## RA delivery systems for immune diseases

### RA mode of action

Inflammation is essential for the regulation of tissue homeostasis and barrier integrity (e.g., BBB, mucosal barrier). However, when dysregulated, it contributes to the pathophysiology of many diseases. In fact, retinoids have been described as potent anti-inflammatory and therefore protective in pathologies such as chronic obstructive pulmonary disease^74^, rheumatoid arthritis^122^, psoriasis^123^, and inflammatory bowel disease^124^. At a cellular level, RA inhibited IL-6-driven induction of proinflammatory Th_17_ cells and promoted the differentiation of T_reg_ cells, which are important to suppress excessive immune responses^125^.

The role of RA in the gastrointestinal tract is particularly relevant since it is produced and metabolized in the intestine, where it regulates the differentiation and function of diverse immune cells and supports mucosal barrier immunity^126^. Indeed, ATRA regulates the activity of CD161^+^-T_reg_ cells that support wound repair in intestinal mucosa^14^. These C-type lectin CD161 regulatory T cells were found to induce cytokines that promoted epithelial barrier healing in the gut. Accordingly, several studies have focused on the impact of vitamin A intake (i.e., RA obtained from the diet), both during development and in the adult. Accordingly, the levels of retinoids obtained from the maternal diet increase the size of secondary lymphoid organs and the efficacy of adult immune responses^13^, while the depletion of vitamin A, in the adult, led to a decrease of Th1, Th17, and ILC3 responses (a synonym of lowered immunity to bacterial infection), an effect which is counteracted through time^127^.

### Type of RA-containing formulations

Several RA formulations have been developed to target immune cells and induce an immunomodulatory response, such as solid lipid nanoparticles^74^, polymeric nanoparticles^58^, nanostructured lipid carriers^124^, among others. RA formulations with an average diameter of 130–250 nm^58,74^ and an entrapment efficiency of ATRA between 2.3 and 310 μg^124^ per mg of nanoparticle have been developed. Macrophage phagocytosis was influenced largely by the physicochemical properties of nanoparticles. For example, uptake is higher in nanoparticles with high negative or positive surface charges^128^. In general, RA formulations showed sustained intracellular RA delivery for a few days^58,74^. RA formulations are taken up by macrophages and induce anti-inflammatory responses by suppressing NF-kB signaling and increasing bone morphogenetic protein 2 signaling (pivotal for bone and cartilage development), as well as by enhancing the production of anti-inflammatory cytokines (e.g., IL-10)^58,124^. In addition, RA formulations have the capacity to enhance the differentiation of naïve T cells to regulatory T cells^124^. The advantages of RA-containing nanoparticles versus free RA were demonstrated by inhibiting expression of IL-6 and IL-8 in alveolar epithelial cells^74^, but were not demonstrated in the induction of immune cell differentiation.

### Preclinical and clinical applications

Clinical trials using free RA in the context of immune thrombocytopenia (severe bleeding disorder) and sclerosing cholangitis (inflammation and scarring of the bile ducts) are active and will evaluate the role of RA as immunotherapy (Table 1). Current challenges for using RA formulations for immune diseases are related to the in vivo demonstration of RA effects. This requires the use of formulations able to target-specific cells, particularly immune cells, and their surface receptors (Fig. 4). There are classes of molecules, including antibodies, carbohydrates, peptides, aptamers that can be attached to RA formulations to target more specific immune cell populations.

## Future outlook

The advantages of RA formulations compared to free RA have been demonstrated in various biomedical applications. These are related to: (1) increased bioavailability; (2) decreased toxicity and skin irritation; (3) increased in vivo half-life; (4) increased RA photostability; (5) increased cell targeting and capacity to cross biological barriers. The advantages of RA formulations have been demonstrated in preclinical (in all applications described in this review) and clinical trials (cancer and skin applications)^71,129^. In the case of cancer, recent studies indicate that the antitumoral activity of RA might be greatly enhanced with formulations that extend the in vivo half-life of RA^12^.

RA formulations have some limitations that preclude a more significant translation to the clinic. For example, ATRA, 9-*cis*-RA, and 13-*cis*-RA, which belong to the first generation of retinoids, are being gradually replaced in some applications by third-generation retinoids^130^. Although many of these formulations have been tested in preclinical models, the clinical translation is still relatively low. For example, although nanoparticles delivered systemically have been extensively explored in animal tests, clinical translation remains compromised because of delivery (e.g., limited efficacy in terms of cell targeting, limited capacity to cross biological barriers), technical (e.g., scale-up), and regulatory aspects (e.g., study design and approval challenges). Use of antibodies, peptides, aptamers immobilized in the surface of nanoparticles to target-specific cellular receptors may accelerate clinical translation of RA formulations. Indeed, several targeted nanoparticle formulations are now being tested in clinical trials for cancer treatment, decorated in their surface with antibodies against transferrin receptor, epidermal growth factor receptor, and ephrin type A receptor 2^131^. In addition, the recent FDA approval of gemtuzumab ozogamicin (an antibody–drug conjugate in which the anti-CD33 antibody is linked to a cytotoxic agent)^106^ or daratumumab (anti-CD38 antibody)^132^ might accelerate clinical testing and the approval of other formulations conjugated with antibodies. Another issue that deserves further attention in the near future is the scale-up of the RA formulations since nanoparticles/microparticles used in most preclinical studies have been prepared in small batches. Scale-up of these formulations is challenging, as they must comply with regulatory guidelines, in terms of narrow size distribution, precise chemical composition, and drug loading^131^. In the last years, progress has been made in producing more controlled formulations with size and composition levels compatible with microfluidic systems^133^, which may advance clinical translation.

Despite steady progress in the last 50 years, many issues remain to be addressed. For example, further preclinical and clinical studies are necessary to evaluate the mechanisms governing the biodistribution, pharmacokinetics, clearance, and toxicology of RA formulations. This requires the development of theranostic RA formulations that may be tracked in vivo by luminescence, magnetic resonance imaging, or positron emission tomography. Theranostic RA formulations are particularly relevant if they are injected intravenously. Moreover, further formulations should be developed with the capacity to release multiple drugs in combination with RA. Pathologies discussed in this review (neurologic diseases, cancer, immune diseases) are multifactorial and display complex signaling pathways and symptoms. Although some progress has been made in the last 5 years regarding formulations with the ability to release RA in combination with other agents^54,112^, further effort is needed to better control the half-life of each drug in vivo to match clinical dosage programs. Another area that deserves further investigation is the development of stimuli-responsive RA formulations that can undergo physical and/or chemical changes in response to endogenous biological or external triggers^64^. The concept here is that the systems retain the drug and release it only after a specific trigger, enhancing its therapeutic efficacy and minimizing systemic toxicity. These systems may be used for the targeted delivery of RA in hematopoietic^25^ or neurogenic niches^60^. Development of these formulations requires the use of linkers susceptible to pH or enzymes present in the targeted cell/tissue. For example, several RA-polymer conjugates have been developed to release RA by hydrolytic cleavage of ester bonds^50,52^, by enzymatic cleavage of amide bonds by proteases present in the targeted tissue^28,65–67^ or by reducing environments^56^ such as the cell cytoplasm. In addition, linkers may be cleaved by an external trigger, such as light with the consequent release of RA^25,26^.

RA is an evolutionarily ancient and conserved molecule with overlapping and pleiotropic effects. Given the diverse pharmacological activities of RA, there is ample potential for developing therapeutic applications. Thus, the fine-tuning of RA-properties and targeted formulation-based delivery is very attractive for customized and more efficient therapies. It is expected that the knowledge gathered in the development of retinoid formulations inspires others to generate delivery systems based on different drugs thus expanding the field of regenerative and therapeutic medicine.

## References

[1] Karrer P, Morf R, Schopp K (1931). Information on vitamine A from train-oil. Helv. Chim. Acta.

[2] Raza K (2013). Nano-lipoidal carriers of tretinoin with enhanced percutaneous absorption, photostability, biocompatibility and anti-psoriatic activity. Int J. Pharm..

[3] Breitman TR, Selonick SE, Collins SJ (1980). Induction of differentiation of the human promyelocytic leukemia cell line (HL-60) by retinoic acid. Proc. Natl Acad. Sci. USA.

[4] Meyskens FL (1983). A phase I trial of beta-all-*trans*-retinoic acid delivered via a collagen sponge and a cervical cap for mild or moderate intraepithelial cervical neoplasia. J. Natl. Cancer Inst..

[5] Graham V, Surwit ES, Weiner S, Meyskens FL (1986). Phase II trial of beta-all-*trans*-retinoic acid for cervical intraepithelial neoplasia delivered via a collagen sponge and cervical cap. West. J. Med..

[6] Chew SY, Hufnagel TC, Lim CT, Leong KW (2006). Mechanical properties of single electrospun drug-encapsulated nanofibres. Nanotechnology.

[7] Carpenedo RL (2009). Homogeneous and organized differentiation within embryoid bodies induced by microsphere-mediated delivery of small molecules. Biomaterials.

[8] Cabezas-Wallscheid N (2017). Vitamin A-retinoic acid signaling regulates hematopoietic stem cell dormancy. Cell.

[9] Chanda B, Ditadi A, Iscove NN, Keller G (2013). Retinoic acid signaling is essential for embryonic hematopoietic stem cell development. Cell.

[10] Farinello D (2018). A retinoic acid-dependent stroma-leukemia crosstalk promotes chronic lymphocytic leukemia progression. Nat. Commun..

[11] Kozono S (2018). Arsenic targets Pin1 and cooperates with retinoic acid to inhibit cancer-driving pathways and tumor-initiating cells. Nat. Commun..

[12] Wei S (2015). Active Pin1 is a key target of all-*trans* retinoic acid in acute promyelocytic leukemia and breast cancer. Nat. Med..

[13] van de Pavert SA (2014). Maternal retinoids control type 3 innate lymphoid cells and set the offspring immunity. Nature.

[14] Povoleri GAM (2018). Human retinoic acid-regulated CD161(+) regulatory T cells support wound repair in intestinal mucosa. Nat. Immunol..

[15] Johansson HJ (2013). Retinoic acid receptor alpha is associated with tamoxifen resistance in breast cancer. Nat. Commun..

[16] Hashimoto-Hill S (2018). RARalpha supports the development of Langerhans cells and langerin-expressing conventional dendritic cells. Nat. Commun..

[17] Chronopoulos A (2016). ATRA mechanically reprograms pancreatic stellate cells to suppress matrix remodelling and inhibit cancer cell invasion. Nat. Commun..

[18] Szuts EZ, Harosi FI (1991). Solubility of retinoids in water. Arch. Biochem. Biophys..

[19] Ourique AF (2011). Improved photostability and reduced skin permeation of tretinoin: development of a semisolid nanomedicine. Eur. J. Pharm. Biopharm..

[20] Adamson PC (1994). Pharmacokinetics of all-*trans*-retinoic acid: clinical implications in acute promyelocytic leukemia. Semin. Hematol..

[21] Pan XQ (2002). Strategy for the treatment of acute myelogenous leukemia based on folate receptor beta-targeted liposomal doxorubicin combined with receptor induction using all-*trans* retinoic acid. Blood.

[22] Han, X. X. et al. Reversal of pancreatic desmoplasia by re-educating stellate cells with a tumour microenvironment-activated nanosystem. *Nat. Commun*. **9**, 3390 10.1038/s41467-018-05906-x (2018). 10.1038/s41467-018-05906-x PMC6107580 30139933

[23] Kong M (2017). Biodegradable hollow mesoporous silica nanoparticles for regulating tumor microenvironment and enhancing antitumor efficiency. Theranostics.

[24] Ferreira R (2016). Retinoic acid-loaded polymeric nanoparticles enhance vascular regulation of neural stem cell survival and differentiation after ischaemia. Nanoscale.

[25] Boto C (2017). Prolonged intracellular accumulation of light-inducible nanoparticles in leukemia cells allows their remote activation. Nat. Commun..

[26] Jimenez-Balsa A (2018). Nanoparticles conjugated with photocleavable linkers for the intracellular delivery of biomolecules. Bioconjug. Chem..

[27] Morales JO, Valdes K, Morales J, Oyarzun-Ampuero F (2015). Lipid nanoparticles for the topical delivery of retinoids and derivatives. Nanomedicine.

[28] Duester G (2008). Retinoic acid synthesis and signaling during early organogenesis. Cell.

[29] Altucci L, Leibowitz MD, Ogilvie KM, de Lera AR, Gronemeyer H (2007). RAR and RXR modulation in cancer and metabolic disease. Nat. Rev. Drug Discov..

[30] di Masi A (2015). Retinoic acid receptors: from molecular mechanisms to cancer therapy. Mol. Asp. Med..

[31] Wagner N, Benkali K, Alio Saenz A, Poncet M, Graeber M (2020). Clinical pharmacology and safety of trifarotene, a first-in-class RARgamma-selective topical retinoid. J. Clin. Pharmacol..

[32] Huen AO, Kim EJ (2015). The role of systemic retinoids in the treatment of cutaneous T-cell lymphoma. Dermatol. Clin..

[33] Gaikwad J, Sharma S, Hatware KV (2020). Review on characteristics and analytical methods of tazarotene: an update. Crit. Rev. Anal. Chem..

[34] Maden M (2007). Retinoic acid in the development, regeneration and maintenance of the nervous system. Nat. Rev. Neurosci..

[35] Schenk T, Stengel S, Zelent A (2014). Unlocking the potential of retinoic acid in anticancer therapy. Br. J. Cancer.

[36] Nunez V (2010). Retinoid X receptor alpha controls innate inflammatory responses through the up-regulation of chemokine expression. Proc. Natl Acad. Sci. USA.

[37] Levin AA, B. T. KazmerS, Grippo JF (1992). 13-cis retinoic acid does not bind to retinoic acid receptors alpha, beta and gamma. Toxicologist.

[38] Al Tanoury Z, Piskunov A, Rochette-Egly C (2013). Vitamin A and retinoid signaling: genomic and nongenomic effects. J. Lipid Res..

[39] Fiorella PD, Napoli JL (1994). Microsomal retinoic acid metabolism. Effects of cellular retinoic acid-binding protein (type I) and C18-hydroxylation as an initial step. J. Biol. Chem..

[40] Stern R (1989). When a uniquely effective drug is teratogenic: the case of isotretinoin. N. Engl. J. Med..

[41] Muindi J (1992). Continuous treatment with all-*trans* retinoic acid causes a progressive reduction in plasma drug concentrations: implications for relapse and retinoid “resistance” in patients with acute promyelocytic leukemia. Blood.

[42] Zanotti G, Dacunto MR, Malpeli G, Folli C, Berni R (1995). Crystal-structure of the transthyretin retinoic-acid complex. Eur. J. Biochem..

[43] Thunemann AF, Beyermann J (2000). Polyethylenimine complexes with retinoic acid: structure, release profiles, and nanoparticles. Macromolecules.

[44] Maia J (2010). Controlling the neuronal differentiation of stem cells by the intracellular delivery of retinoic acid-loaded nanoparticles. ACS Nano.

[45] Zhang R (2016). Traceable nanoparticle delivery of small interfering RNA and retinoic acid with temporally release ability to control neural stem cell differentiation for Alzheimer’s disease therapy. Adv. Mater..

[46] Giordano GG, Refojo MF, Arroyo MH (1993). Sustained delivery of retinoic acid from microspheres of biodegradable polymer in PVR. Investig. Ophthalmol. Vis. Sci..

[47] Jeong YI (2006). Polyion complex micelles composed of all-*trans* retinoic acid and poly (ethylene glycol)-grafted-citosan. J. Pharm. Sci..

[48] Kim DG, Jeong YI, Nah JW (2007). All-*trans* retinoic acid release from polyion-complex micelles of methoxy poly(ethylene glycol) grafted chitosan. J. Appl. Polym. Sci..

[49] Mehta K, Sadeghi T, Mcqueen T, Lopezberestein G (1994). Liposome encapsulation circumvents the hepatic-clearance mechanisms of all-*trans*-retinoic acid. Leuk. Res..

[50] Nam YS (2003). Chemical immobilization of retinoic acid within poly(epsilon-caprolactone) nanoparticles based on drug-polymer bioconjugates. J. Appl. Polym. Sci..

[51] Kim DG (2006). All-*trans* retinoic acid-associated low molecular weight water-soluble chitosan nanoparticles based on ion complex. Macromol. Res..

[52] Castleberry SA, Quadir MA, Abu Sharkh M, Shopsowitz KE, Hammond PT (2017). Polymer conjugated retinoids for controlled transdermal delivery. J. Control. Release.

[53] Hou L, Yao J, Zhou JP, Zhang Q (2012). Pharmacokinetics of a paclitaxel-loaded low molecular weight heparin-all-*trans*-retinoid acid conjugate ternary nanoparticulate drug delivery system. Biomaterials.

[54] Yao J, Zhang L, Zhou JP, Liu HP, Zhang Q (2013). Efficient simultaneous tumor targeting delivery of all-trans retinoid acid and paclitaxel based on hyaluronic acid-based multifunctional nanocarrier. Mol. Pharm..

[55] Park KM (2009). All-*trans*-retinoic acid (ATRA)-grafted polymeric gene carriers for nuclear translocation and cell growth control. Biomaterials.

[56] Huang H (2017). Co-delivery of all-*trans*-retinoic acid enhances the anti-metastasis effect of albumin-bound paclitaxel nanoparticles. Chem. Commun..

[57] Cho CS (2001). Receptor-mediated delivery of all trans-retinoic acid to hepatocyte using poly(L-lactic acid) nanoparticles coated with galactose-carrying polystyrene. J. Control. Release.

[58] Almouazen E (2012). Development of a nanoparticle-based system for the delivery of retinoic acid into macrophages. Int J. Pharm..

[59] Park SJ (2017). Highly efficient and rapid neural differentiation of mouse embryonic stem cells based on retinoic acid encapsulated porous nanoparticle. ACS Appl. Mater. Interfaces.

[60] Santos T (2017). Blue light potentiates neurogenesis induced by retinoic acid-loaded responsive nanoparticles. Acta Biomater..

[61] Choi Y (2001). Long-term delivery of all-*trans*-retinoic acid using biodegradable PLLA/PEG-PLLA blended microspheres. Int. J. Pharm..

[62] Jeong YI (2006). Polyion complex micelles composed of all-*trans* retinoic acid and poly (ethylene glycol)-grafted-chitosan. J. Pharm. Sci..

[63] Zuccari G, Carosio R, Fini A, Montaldo PG, Orienti I (2005). Modified polyvinylalcohol for encapsulation of all-*trans*-retinoic acid in polymeric micelles. J. Control. Release.

[64] Mura S, Nicolas J, Couvreur P (2013). Stimuli-responsive nanocarriers for drug delivery. Nat. Mater..

[65] Schug TT, Berry DC, Shaw NS, Travis SN, Noy N (2007). Opposing effects of retinoic acid on cell growth result from alternate activation of two different nuclear receptors. Cell.

[66] Santos T (2012). Polymeric nanoparticles to control the differentiation of neural stem cells in the subventricular zone of the brain. ACS Nano.

[67] Muindi JR (1992). Clinical pharmacology of oral all-*trans* retinoic acid in patients with acute promyelocytic leukemia. Cancer Res..

[68] Esteves M (2015). Retinoic acid-loaded polymeric nanoparticles induce neuroprotection in a mouse model for Parkinson’s disease. Front. Aging Neurosci..

[69] Nastruzzi C, Walde P, Menegatti E, Gambari R (1990). Liposome-associated retinoic acid. Increased in vitro antiproliferative effects on neoplastic cells. FEBS Lett..

[70] Estey E (1996). Alterations in tretinoin pharmacokinetics following administration of liposomal all-*trans* retinoic acid. Blood.

[71] Rahman SA (2016). Tretinoin-loaded liposomal formulations: from lab to comparative clinical study in acne patients. Drug Deliv..

[72] Janesick A, Wu SC, Blumberg B (2015). Retinoic acid signaling and neuronal differentiation. Cell. Mol. Life Sci..

[73] Tzezana R, Reznik S, Blumenthal J, Zussman E, Levenberg S (2012). Regulation of stem cell differentiation by control of retinoic acid gradients in hydrospun 3D scaffold. Macromol. Biosci..

[74] Payne CM (2019). Evaluation of the immunomodulatory effects of all-*trans* retinoic acid solid lipid nanoparticles and human mesenchymal stem cells in an A549 epithelial cell line model. Pharm. Res..

[75] Sardana K, Sehgal VN (2003). Retinoids: fascinating up-and-coming scenario. J. Dermatol..

[76] Saiag P (1998). Treatment of early AIDS-related Kaposi’s sarcoma with oral all-*trans-*retinoic acid: results of a sequential non-randomized phase II trial. Aids.

[77] Castro GA (2009). Formation of ion pairing as an alternative to improve encapsulation and stability and to reduce skin irritation of retinoic acid loaded in solid lipid nanoparticles. Int J. Pharm..

[78] Masini V, Bonte F, Meybeck A, Wepierre J (1993). Cutaneous bioavailability in hairless rats of tretinoin in liposomes or gel. J. Pharm. Sci..

[79] Mehnert W, Mader K (2012). Solid lipid nanoparticles production, characterization and applications. Adv. Drug Deliv. Rev..

[80] Ourique AF, Pohlmann AR, Guterres SS, Beck RCR (2008). Tretinoin-loaded nanocapsules: preparation, physicochemical characterization, and photostability study. Int J. Pharm..

[81] Lapteva M, Moller M, Gurny R, Kalia YN (2015). Self-assembled polymeric nanocarriers for the targeted delivery of retinoic acid to the hair follicle. Nanoscale.

[82] Xue J (2017). Neutrophil-mediated anticancer drug delivery for suppression of postoperative malignant glioma recurrence. Nat. Nanotechnol..

[83] Zlokovic BV (2011). Neurovascular pathways to neurodegeneration in Alzheimer’s disease and other disorders. Nat. Rev. Neurosci..

[84] Ding Y (2008). Retinoic acid attenuates beta-amyloid deposition and rescues memory deficits in an Alzheimer’s disease transgenic mouse model. J. Neurosci..

[85] Chakrabarti M (2016). Molecular signaling mechanisms of natural and synthetic retinoids for inhibition of pathogenesis in Alzheimer’s disease. J. Alzheimers Dis..

[86] McCaffery P, Dräger U (1994). High levels of a retinoic acid-generating dehydrogenase in the meso-telencephalic dopamine system. Proc. Natl Acad. Sci..

[87] Jankovic J, Chen S, Le W (2005). The role of Nurr1 in the development of dopaminergic neurons and Parkinson’s disease. Prog. Neurobiol..

[88] Smits SM, Ponnio T, Conneely OM, Burbach JPH, Smidt MP (2003). Involvement of Nurr1 in specifying the neurotransmitter identity of ventral midbrain dopaminergic neurons. Eur. J. Neurosci..

[89] Islam MM, Mohamed Z (2015). Computational and pharmacological target of neurovascular unit for drug design and delivery. Biomed. Res. Int..

[90] Mizee MR (2013). Retinoic acid induces blood-brain barrier development. J. Neurosci..

[91] Machado-Pereira M, Santos T, Ferreira L, Bernardino L, Ferreira R (2018). Intravenous administration of retinoic acid-loaded polymeric nanoparticles prevents ischemic injury in the immature brain. Neurosci. Lett..

[92] Burnett AK (2015). Arsenic trioxide and all-*trans* retinoic acid treatment for acute promyelocytic leukaemia in all risk groups (AML17): results of a randomised, controlled, phase 3 trial. Lancet Oncol..

[93] Drach J, Lopezberestein G, Mcqueen T, Andreeff M, Mehta K (1993). Induction of differentiation in myeloid-leukemia cell-lines and acute promyelocytic leukemia-cells by liposomal all-*trans*-retinoic acid. Cancer Res..

[94] Ezpeleta I (1996). Gliadin nanoparticles for the controlled release of all-*trans*-retinoic acid. Int J. Pharm..

[95] Estey EH (1999). Molecular remissions induced by liposomal-encapsulated all-*trans* retinoic acid in newly diagnosed acute promyelocytic leukemia. Blood.

[96] Mu CF (2018). Targeted drug delivery for tumor therapy inside the bone marrow. Biomaterials.

[97] Duan CW (2014). Leukemia propagating cells rebuild an evolving niche in response to therapy. Cancer Cell.

[98] Montesinos P (2009). Differentiation syndrome in patients with acute promyelocytic leukemia treated with all-*trans* retinoic acid and anthracycline chemotherapy: characteristics, outcome, and prognostic factors. Blood.

[99] Russo D (1998). All-*trans* retinoic acid (ATRA) in patients with chronic myeloid leukemia in the chronic phase. Leukemia.

[100] Swami A (2014). Engineered nanomedicine for myeloma and bone microenvironment targeting. Proc. Natl Acad. Sci. USA.

[101] Lu Y (2007). Role of formulation composition in folate receptor-targeted liposomal doxorubicin delivery to acute myelogenous leukemia cells. Mol. Pharm..

[102] Stephan MT, Moon JJ, Um SH, Bershteyn A, Irvine DJ (2010). Therapeutic cell engineering with surface-conjugated synthetic nanoparticles. Nat. Med..

[103] Dorrance AM (2015). Targeting leukemia stem cells in vivo with antagomiR-126 nanoparticles in acute myeloid leukemia. Leukemia.

[104] Barth BM (2011). Targeted indocyanine-green-loaded calcium phosphosilicate nanoparticles for in vivo photodynamic therapy of leukemia. ACS Nano.

[105] Zong H (2016). In vivo targeting of leukemia stem cells by directing parthenolide-loaded nanoparticles to the bone marrow niche. Leukemia.

[106] Abdel-Wahab, O. A landmark year for FDA-approved therapies for acute myeloid leukemia. *Blood* **15**10.1002/phar.2180 (2018).

[107] Meyskens FL (1994). Enhancement of regression of cervical intraepithelial neoplasia-ii (moderate dysplasia) with topically applied all-*trans*-retinoic acid—a randomized trial. J. Natl. Cancer Inst..

[108] Hua SJ, Kittler R, White KP (2009). Genomic antagonism between retinoic acid and estrogen signaling in breast cancer. Cell.

[109] Sutton LM, Warmuth MA, Petros WP, Winer EP (1997). Pharmacokinetics and clinical impact of all-*trans* retinoic acid in metastatic breast cancer: a phase II trial. Cancer Chemother. Pharmacol..

[110] Budd GT (1998). Phase I/II trial of all-*trans* retinoic acid and tamoxifen in patients with advanced breast cancer. Clin. Cancer Res..

[111] Bryan M (2011). A pilot phase II trial of all-*trans* retinoic acid (Vesanoid) and paclitaxel (Taxol) in patients with recurrent or metastatic breast cancer. Investig. New Drugs.

[112] Zhang Y (2016). Retinal-conjugated pH-sensitive micelles induce tumor senescence for boosting breast cancer chemotherapy. Biomaterials.

[113] Li RJ (2011). All-*trans* retinoic acid stealth liposomes prevent the relapse of breast cancer arising from the cancer stem cells. J. Control. Release.

[114] Sun R (2015). Co-delivery of all-*trans*-retinoic acid and doxorubicin for cancer therapy with synergistic inhibition of cancer stem cells. Biomaterials.

[115] See SJ, Levin VA, Yung WK, Hess KR, Groves MD (2004). 13-cis-retinoic acid in the treatment of recurrent glioblastoma multiforme. Neuro Oncol..

[116] Matthay KK (2009). Long-term results for children with high-risk neuroblastoma treated on a randomized trial of myeloablative therapy followed by 13-cis-retinoic acid: a children’s oncology group study. J. Clin. Oncol..

[117] Ying M (2011). Regulation of glioblastoma stem cells by retinoic acid: role for Notch pathway inhibition. Oncogene.

[118] Niu CS (2010). Effect of all-*trans* retinoic acid on the proliferation and differentiation of brain tumor stem cells. J. Exp. Clin. Cancer Res..

[119] Karsy M, Albert L, Tobias ME, Murali R, Jhanwar-Uniyal M (2010). All-*trans* retinoic acid modulates cancer stem cells of glioblastoma multiforme in an MAPK-dependent manner. Anticancer Res..

[120] Liu JL (2017). Preparation of N, N, N-trimethyl chitosan-functionalized retinoic acid-loaded lipid nanoparticles for enhanced drug delivery to glioblastoma. Trop. J. Pharm. Res..

[121] Wang W (2016). Effector T cells abrogate stroma-mediated chemoresistance in ovarian cancer. Cell.

[122] Nozaki Y (2006). Anti-inflammatory effect of all-*trans*-retinoic acid in inflammatory arthritis. Clin. Immunol..

[123] Fredriksson T, Pettersson U (1978). Severe psoriasis—oral therapy with a new retinoid. Dermatologica.

[124] Zai K (2019). Regulation of inflammatory response of macrophages and induction of regulatory T cells by using retinoic acid-loaded nanostructured lipid carrier. J. Biomater. Sci. Polym. Ed..

[125] Mucida D (2007). Reciprocal T(H)17 and regulatory T cell differentiation mediated by retinoic acid. Science.

[126] Erkelens MN, Mebius RE (2017). Retinoic acid and immune homeostasis: a balancing act. Trends Immunol..

[127] Spencer SP (2014). Adaptation of innate lymphoid cells to a micronutrient deficiency promotes type 2 barrier immunity. Science.

[128] He CB, Hu YP, Yin LC, Tang C, Yin CH (2010). Effects of particle size and surface charge on cellular uptake and biodistribution of polymeric nanoparticles. Biomaterials.

[129] Jain P (2014). Single-agent liposomal all-*trans*-retinoic acid as initial therapy for acute promyelocytic leukemia: 13-year follow-up data. Clin. Lymphoma Myeloma Leuk..

[130] Leyden J, Stein-Gold L, Weiss J (2017). Why topical retinoids are mainstay of therapy for acne. Dermatol. Ther..

[131] Anselmo AC, Mitragotri S (2019). Nanoparticles in the clinic: an update. Bioeng. Transl. Med..

[132] Deshantri AK (2018). Nanomedicines for the treatment of hematological malignancies. J. Control. Release.

[133] Karnik R (2008). Microfluidic platform for controlled synthesis of polymeric nanoparticles. Nano Lett..

[134] Shelley RS, Jun HW, Price JC, Cadwallader DE (1982). Blood level studies of all-*trans*-retinoic and 13-Cis-retinoic acids in rats using different formulations. J. Pharm. Sci..

[135] Puppi D, Piras AM, Detta N, Dinucci D, Chiellini F (2010). Poly(lactic-co-glycolic acid) electrospun fibrous meshes for the controlled release of retinoic acid. Acta Biomater..

[136] O’Leary C, O’Brien FJ, Cryan SA (2017). Retinoic acid-loaded collagen-hyaluronate scaffolds: a bioactive material for respiratory tissue regeneration. ACS Biomater. Sci. Eng..

[137] Damanik FFR, van Blitterswijk C, Rotmans J, Moroni L (2018). Enhancement of synthesis of extracellular matrix proteins on retinoic acid loaded electrospun scaffolds. J. Mater. Chem. B.

[138] Mukherjee S (2006). Retinoids in the treatment of skin aging: an overview of clinical efficacy and safety. Clin. Interv. Aging.

[139] Riahi RR, Bush AE, Cohen PR (2016). Topical retinoids: therapeutic mechanisms in the treatment of photodamaged skin. Am. J. Clin. Dermatol..

[140] Berger R (2007). Tretinoin gel microspheres 0.04% versus 0.1% in adolescents and adults with mild to moderate acne vulgaris: a 12-week, multicenter, randomized, double-blind, parallel-group, phase IV trial. Clin. Ther..

[141] Kligman AM (1998). The growing importance of topical retinoids in clinical dermatology: a retrospective and prospective analysis. J. Am. Acad. Dermatol..

